# TAK1 protein kinase activity is required for TLR signalling and cytokine production in myeloid cells

**DOI:** 10.1042/BCJ20220314

**Published:** 2022-09-16

**Authors:** Melissa Rodrigues, Tsvetana Petrova, Brendan Tibbs, J. Simon C. Arthur, Philip Cohen

**Affiliations:** 1 MRC Protein Phosphorylation and Ubiquitylation Unit, School of Life Sciences, University of Dundee, Dundee, Scotland; 2 Division of Cell Signalling and Immunology, School of Life Sciences, University of Dundee, Dundee, Scotland

**Keywords:** immune cell signaling, MyD88, toll-like receptors

## Abstract

A conditional knock-in mouse was generated in which the TAK1 catalytic subunit was largely replaced by the kinase-inactive TAK1[D175A] mutant in immune cells. The activation of p38α MAP kinase, c-Jun N-terminal kinases 1 and 2 (JNK1/2) and the canonical IKK complex induced by stimulation with several TLR-activating ligands was reduced in bone marrow-derived macrophages (BMDM) from TAK1[D175A] mice. TLR signalling in TAK1[D175A] BMDM was catalysed by the residual wild-type TAK1 in these cells because it was abolished by either of two structurally unrelated TAK1 inhibitors (NG25 and 5Z-7-oxozeaenol) whose off-target effects do not overlap. The secretion of inflammatory mediators and production of the mRNAs encoding these cytokines induced by TLR ligation was greatly reduced in peritoneal neutrophils or BMDM from TAK1[D175A] mice. The Pam3CSK4- or LPS-stimulated activation of MAP kinases and the canonical IKK complex, as well as cytokine secretion, was also abolished in TAK1 knock-out human THP1 monocytes or macrophages. The results establish that TAK1 protein kinase activity is required for TLR-dependent signalling and cytokine secretion in myeloid cells from mice. We discuss possible reasons why other investigators, studying myeloid mice with a conditional knock-out of TAK1 or a different conditional kinase-inactive knock-in of TAK1, reported TAK1 to be a negative regulator of LPS-signalling and cytokine production in mouse macrophages and neutrophils.

## Introduction

Components of microbial pathogens, termed Pathogen Associated Molecular Patterns (PAMPs), activate Toll-Like Receptors (TLRs) in immune cells, switching on signalling networks that control the production and secretion of inflammatory mediators. The interaction of PAMPs with most TLRs or the interaction of proinflammatory cytokines of the Interleukin-1 (IL-1) family (IL-1, IL-18 and IL-33) with their receptors [1] initiates signalling by recruitment of the protein product of myeloid differentiation primary response gene 88 (MyD88), which is followed by the recruitment of Interleukin Receptor-Associated Kinase (IRAK) family members to form oligomeric complexes, termed Myddosomes [2,3].

IRAK1 and IRAK2 interact with and induce the oligomerisation of TNF-Receptor-Associated Factor 6 (TRAF6), which activates the E3 ligase activity of TRAF6 [4–7]. IRAK1 additionally phosphorylates and activates the E3 ligases Pellino 1 and Pellino 2 [8,9]. Together, TRAF6 and Pellino1/2 produce the Lys63-linked ubiquitin (K63-Ub) chains [8,10,11] that bind to the TAB2 and TAB3 components of the TAK1 kinase complexes TAB1-TAK1-TAB2 [12] and TAB1-TAK1-TAB3 [13] inducing conformational changes that activate TAK1 (also called MAP3K7). TAK1 phosphorylates and activates the mitogen-activated protein kinase kinases (MAPK kinases) that switch on p38α MAPK (MKK3, MKK4 and MKK6) and JNK1/2 (MKK4 and MKK7) [14] and initiate activation of the canonical IKK complex [15]. Together, these protein kinases catalyse many phosphorylation events that drive the production of inflammatory mediators.

The essential role of the TAK1 protein kinase activity in IL-1, TNF and LPS signalling has been established in embryonic fibroblasts, and in TLR and CD40 stimulated B cells, by studying cells derived from knock-in mice expressing a truncated, inactive form of TAK1 lacking amino acid residues 40–78 in the ATP binding site of the catalytic subunit (TAK1[Δ40–78]) [16]. IL-1 signalling is also abolished in TAK1 KO human embryonic kidney (HEK) 293 cells and TAK1 KO HaCaT cells, an immortalised human keratinocyte cell line, and restored by re-transfection of WT TAK1 but not by the kinase-inactive TAK1[D175A] mutant (Supplementary Figure S1C in [10]).

TAK1 KO mice die at an early embryonic stage [16–18]. Therefore, to investigate the role of TAK1 in myeloid cells, conditional TAK1 knock-out mice were crossed to LyzM-Cre mice (also called Lyz2-Cre) to generate mice deficient in TAK1 in myeloid cells (TAK1 KO × LyzM-Cre mice). The expression of TAK1 was reduced (but not abolished) in thioglycolate-elicited peritoneal macrophages and neutrophils from these mice [19]. The mice displayed enlarged lymph nodes, an enlarged spleen and increased numbers of splenic and bone marrow neutrophils. In contrast with TAK1-deficient MEFs in which LPS-stimulated activation of p38α MAPK, JNK1/2 and the canonical IKK complex was abolished, the LPS-stimulated activation of these protein kinases was little affected in macrophages and enhanced in neutrophils from TAK1 KO × LyzM-Cre mice. Taken together, these results suggested that, in contrast with other cells studied previously, TAK1 was not essential for LPS-signalling and cytokine production in macrophages and was a negative regulator of LPS signalling and cytokine production in neutrophils.

Other investigators crossed the kinase-inactive TAK1[Δ40–78] mutant to LyzM-Cre mice to generate TAK1[Δ40–78] × LyzM-Cre mice. Based on Southern blotting analysis, ∼50% of the WT TAK1 was replaced by the TAK1[Δ40–78] mutant in the bone marrow-derived macrophages (BMDM) derived from these mice [20]. The LPS-stimulated activation of JNK1/2, the binding of NF-κB to DNA and the secretion of IL-10 were decreased in BMDM from TAK1[Δ40–78] × LyzM-Cre mice, but the LPS-stimulated secretion of IL-1β, IL-6 and TNF was unaffected or slightly increased in BMDM and increased significantly in neutrophils from TAK1[Δ40–78] × LyzM-Cre mice. In contrast with TAK1 KO × LyzM-Cre mice, the TAK1[Δ40–78] × LyzM-Cre mice did not show any gross morphological changes in the overall histology of their lymphoid organs.

Here, we re-investigated the role of TAK1 catalytic activity in macrophages and neutrophils using a conditional TAK1[D175A] knock-in mouse crossed to mice expressing the Vav-iCre transgene (TAK1[D175A] × Vav-iCre mice). Asp175 is located in the ‘DFG’ motif, which binds to the Mg^2+^ ion of the Mg-ATP substrate in the ATP-binding site, and its mutation to Ala generates a kinase-inactive mutant without altering the kinase conformation significantly [21]. Our results establish that, as in other cells, TAK1 catalytic activity is required for TLR signalling and cytokine production in both BMDM and neutrophils. We discuss possible reasons for the different results and conclusions reached previously by other laboratories.

## Results

### Generation of conditional knock-in mice expressing the kinase-inactive TAK1[D175A] mutant

Mice with the conditional TAK1[D175A] mutation were generated as described in Methods and crossed with mice expressing the Vav-iCre transgene to replace WT TAK1 with the kinase-inactive TAK1[D175A] mutant in immune cells (Supplementary Figure S1). RNA from the blood of these mice was analysed by qPCR using TaqMan probes specific for either the wild type (WT) or D175A sequence. These experiments showed that the replacement of WT TAK1 mRNA by the TAK1[D175A] mRNA was incomplete, varying from 33% to 99% TAK1[D175A] mRNA for 38 mice that were genotyped as homozygous animals. Only mice that expressed 80–99% of the TAK1[D175A] mutant sequence (20 of the 38 animals) were used in this study.

**Figure 1.**
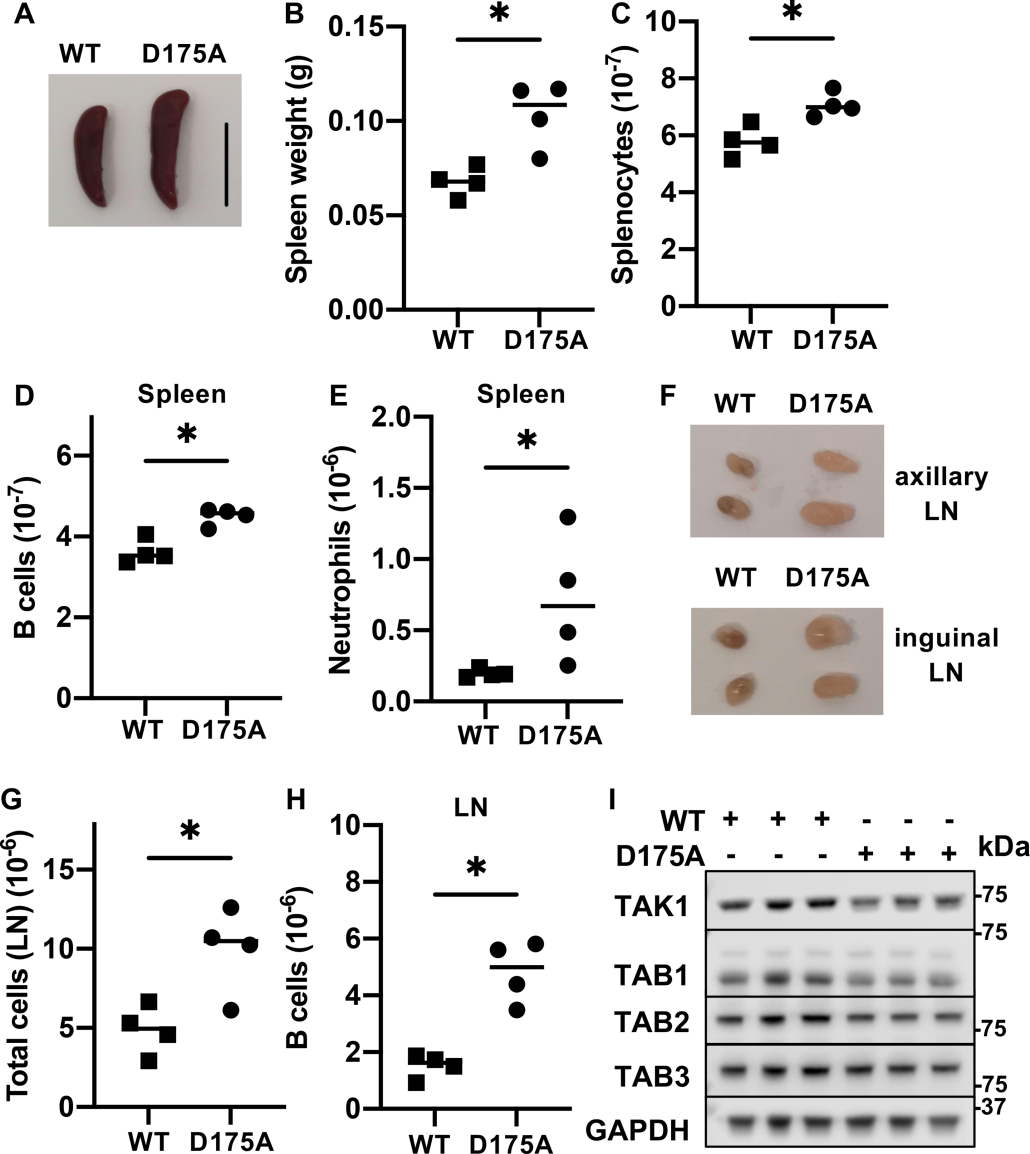
TAK1[D175A] × Vav-iCre mice have enlarged lymphoid organs, increased B cell and neutrophil numbers and normal expression of TAK1 and TAB proteins. (**A**) Representative image of spleens of 12 week old WT and TAK1[D175A] × Vav-iCre mice (D175A), scale bar = 1 cm. (**B**) Spleen weights of 12 week old WT (*n* = 4) and TAK1[D175A] × Vav-iCre mice (*n* = 4). Each symbol represents an individual mouse. (**C**–**E**) As in (**B**) except that splenocyte (**C**), B cell (**D**) and neutrophil (**E**) numbers in the spleen are shown. (**F**) Representative images of axillary (upper panel) and inguinal (lower panel) lymph nodes (LN) of 12 week old WT and TAK1[D175A] × Vav-iCre mice (D175A). (**G**) Total cell numbers in axillary and inguinal LN of 12 week old WT (*n* = 4) and TAK1[D175A] × Vav-iCre mice (*n* = 4). (**H**) As in (**G**), except B cell numbers in the LN are shown. Statistical significance between the two genotypes was calculated using the unpaired *t*-test with Welch's correction; * denotes *P* < 0.05. (**I**) Extracts of BMDM (10 µg protein) from three separate WT mice or three separate TAK1[D175A] × Vav-iCre mice (D175A) (+,+,+) were denatured in SDS, subjected to SDS–PAGE, transferred to PVDF membranes and immunoblotted using the ECL detection system (GE Healthcare) with antibodies recognising TAK1, TAB1, TAB2, TAB3 and GAPDH as a loading control.

The TAK1[D175A] × Vav-iCre mice did not show any obvious deviation from normal welfare up to six months of age when maintained under standard laboratory conditions, but when culled at aged 3 months were found to have enlarged spleens (Figure 1A,B) with an increased number of splenocytes (Figure 1C), splenic B cells (Figure 1D) and splenic neutrophils (Figure 1E). The size of the axillary and inguinal lymph nodes (LN) (Figure 1F) was increased, but not as strikingly as in the TAK1 KO × LyzM-Cre mice [19]. Consistent with the increased size of the lymph nodes in TAK1[D175A] × Vav-iCre mice, lymphocyte numbers (Figure 1G) were also increased, resulting from a higher number of B cells (Figure 1H) without a significant increase in T cell numbers (Supplementary Figure S2).

The bone marrow of TAK1[D175A] × Vav-iCre mice could not be differentiated into macrophages with macrophage colony stimulator factor (M-CSF), but macrophages could be generated using L929 conditioned medium. The number of macrophages generated from the bone marrow of TAK1[D175A] × Vav-iCre mice was only about half the number generated from WT bone marrow (Supplementary Figure S3A), which could be due to a lower rate of proliferation or an increased rate of cell death. We found that the rate of apoptosis was slightly greater in BMDM from the TAK1[D175A] × Vav-iCre mice (Supplementary Figure S3B,C). The BMDM from TAK1[D175A] mice expressed similar levels of the TAK1 protein and its regulatory subunits to BMDM from WT mice (Figure 1I).

### Reduced TLR-stimulated cytokine production in BMDM and peritoneal neutrophils from TAK1[D175A] × Vav-iCre mice

**Figure 2.**
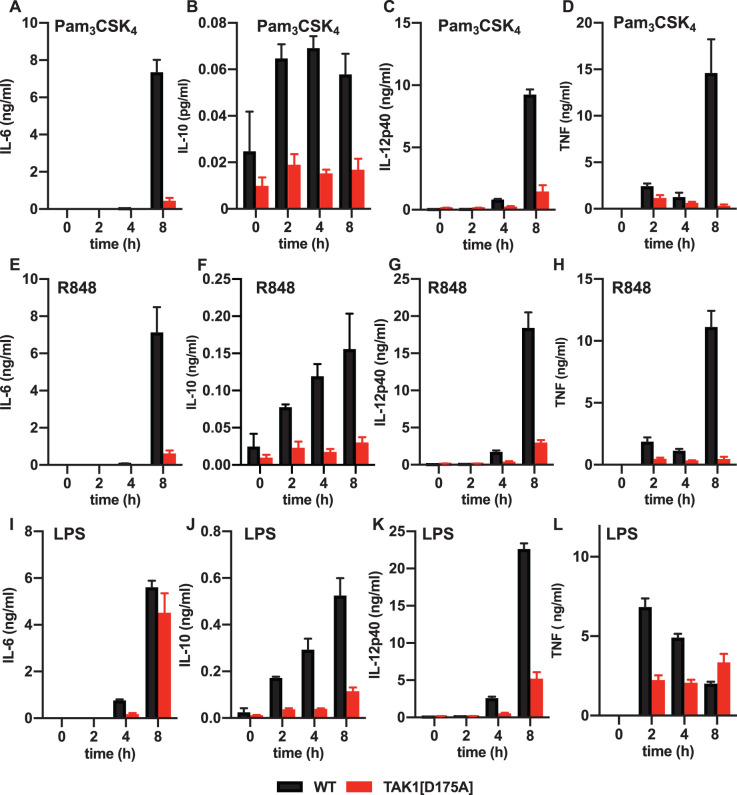
Pam_3_CSK_4_, R848 or LPS-stimulated cytokine secretion is greatly reduced in TAK1[D175A] BMDM. BMDM from 4 WT (black bars) and 4 TAK1[D175A] (red bars) mice were stimulated for the times indicated with 1 µg/ml Pam_3_CSK_4_ (**A**–**D**), 250 ng/ml R848 (**E**–**H**) or 100 ng/ml LPS (**I**–**L**) and the IL-6 (**A**, **E** and **I**), IL-10 (**B**, **F** and **J**), IL-12p40 (**C**, **G** and **K**) and TNF (**D**, **H** and **L**) secreted into the culture medium were measured. Bar graphs show mean values and error bars ± standard error of the mean (SEM). Similar results were obtained in eight other independent experiments comparing BMDM from one WT and one TAK1[D175A] mouse.

**Figure 3.**
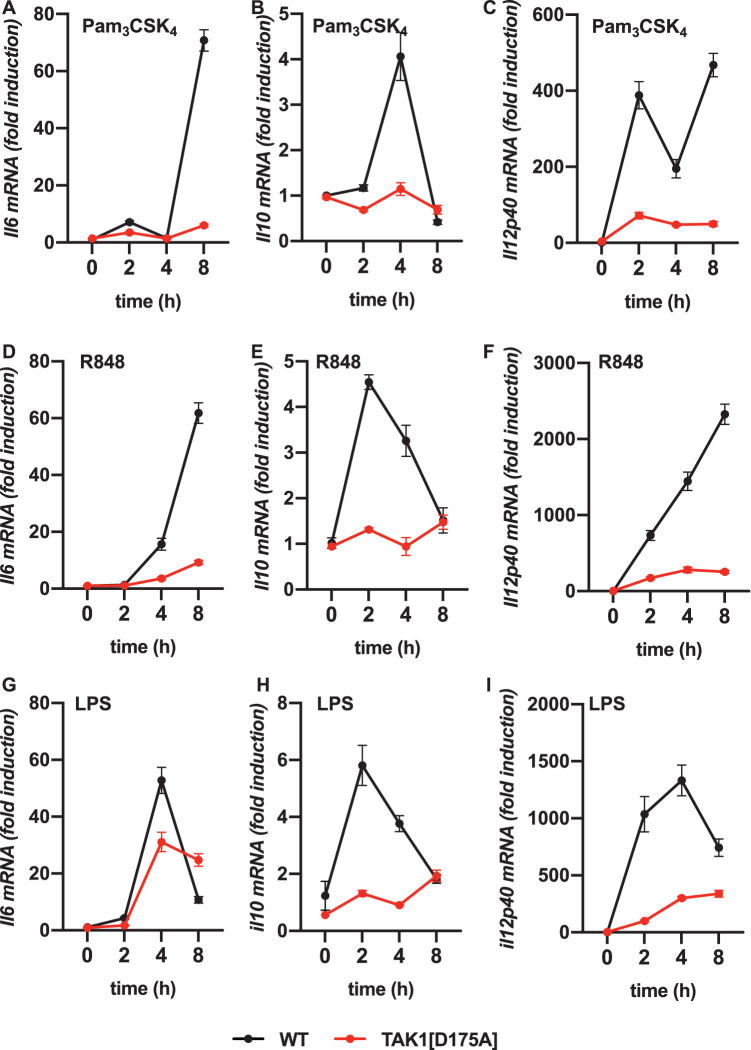
Pam3CSK4, R848 and LPS-stimulated cytokine mRNA levels is greatly reduced in TAK1[D175A] BMDM. BMDMs from 4 WT mice (black circles) and 4 TAK1[D175A] mice (red circles) were stimulated for the times indicated with 1 µg/ml Pam3CSK4 (**A**–**C**), 250 ng/ml R848 (**D**–**F**) or 100 ng/ml LPS (**G**–**I**). RNA was extracted from the cells and *il6* mRNA (**A**, **D**, **G**), *il10* mRNA (**B**, **E**, **H**) and *il12p40* mRNA (**C**, **F**, **I**) and levels were measured by quantitative real-time PCR. Results are presented as fold-increase in mRNA relative to the levels in unstimulated samples. Values were normalised to *GAPDH* mRNA levels. Similar results were obtained in three separate experiments.

We stimulated BMDM from WT and TAK1[D175A] × Vav-iCre mice with Pam3CSK4, an activator of the TLR1/2 heterodimer or R848, an activator of the TLR7/8 heterodimer, which signal exclusively via the adaptor MyD88. We found that the secretion of IL-6, IL-10, IL-12p40 and TNF induced by these ligands was reduced in BMDM from TAK1[D175A] × Vav-iCre mice up to 8 h (Figure 2A–H). Consistent with these results, the production of *il6*, *il10* and *il12p40* mRNA was also greatly reduced in BMDM from TAK1[D175A] × Vav-iCre mice (Figure 3A–F).

We also stimulated BMDM with LPS, an activator of TLR4, which signals via the TIR-domain-containing adapter-inducing interferon-β (TRIF) as well as via MyD88. Similar to Pam3CSK4 and R848, the LPS-induced secretion of IL-10 and IL-12p40 was greatly reduced in BMDM from TAK1[D175A] × Vav-iCre mice up to 8 h (Figure 2J,K). The LPS-induced secretion of IL-6 and TNF was also reduced after stimulation for 4 h, but the secretion of these cytokines became similar after 8 h (Figure 2I,L). Consistent with these findings, the LPS-stimulated production of *il10* and *il12p40* mRNA was greatly reduced in BMDM from TAK1[D175A] × Vav-iCre mice, but the *il6* mRNA was not (Figure 3G–I). These observations are considered further in the Discussion.

**Figure 4.**
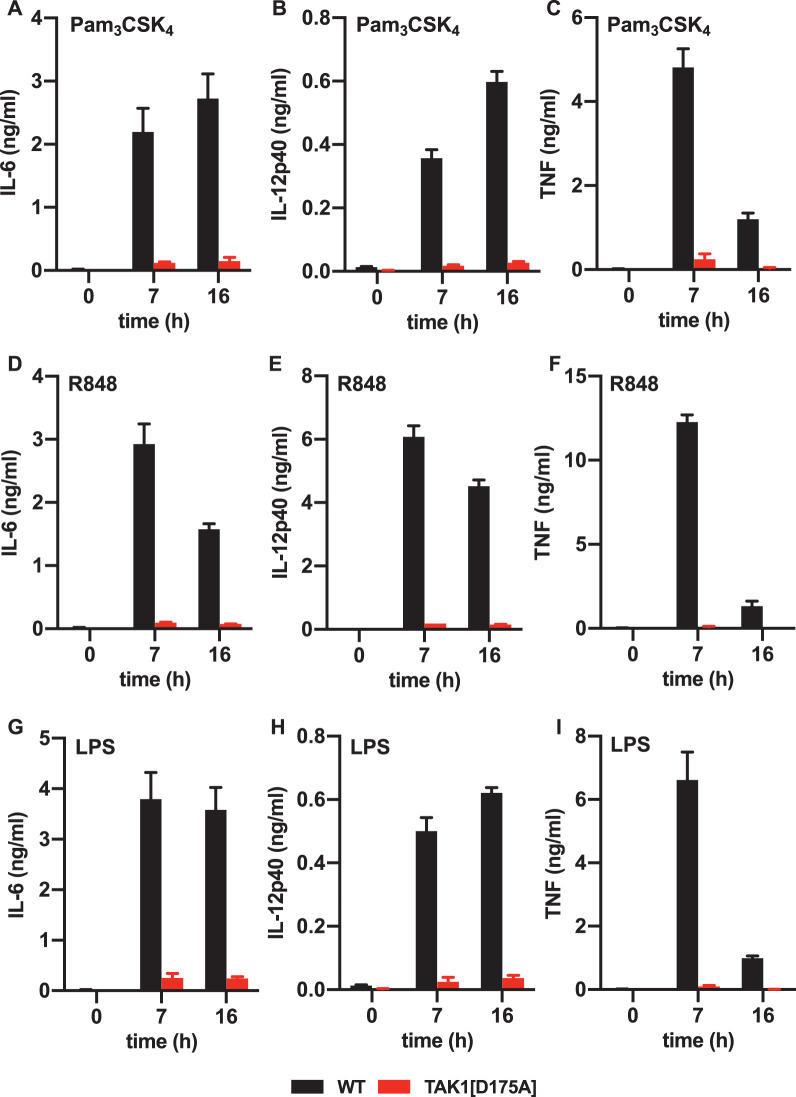
Pam3CSK4, R848 and LPS-stimulated cytokine production is suppressed in TAK1[D175A] neutrophils. Peritoneal neutrophils from WT (black bars) and TAK1[D175A] (red bars) mice were stimulated for the times indicated with 1 µg/ml Pam3CSK4 (**A**–**C**), 250 ng/ml R848 (**D**–**F**) or 100 ng/ml LPS (**G**–**I**). The IL-6 (**A**, **D** and **G**), IL-12p40 (**B**, **E** and **H**) and TNF (**C**, **F** and **I**) secreted into the culture medium was then measured at the times indicated. The results were obtained with the neutrophils from one WT and one TAK1[D175A] × Vav-iCre mice and the figures show the error bars ± standard error of the mean (SEM) for triplicate determinations. Similar results were obtained in four additional independent experiments each comparing neutrophils from one WT and one TAK1[D175A] mouse.

We also studied the effect of the same three TLR ligands on cytokine production in peritoneal neutrophils. The secretion of IL-6, IL-12 and TNF induced by Pam3CSK, R848 or LPS was greatly reduced in peritoneal neutrophils from TAK1[D175A] × Vav-iCre mice at 7 h or 16 h after stimulation (Figure 4).

### Flow cytometry analysis of TNF production in BMDM populations from TAK1[D175A] × Vav-iCre and WT mice

BMDM from three individual TAK1[D175A] × Vav-iCre mice in which the extent of knock-in of the kinase-inactive TAK1 mutant averaged 91% (Supplementary Figure S4A) were used for these studies. As expected, they produced reduced amounts of TNF compared with BMDM from WT littermates (Supplementary Figure S4B), but further analysis by flow cytometry revealed that although most of the WT BMDM produced TNF in response to TLR ligands, the response of the TAK1[D175A] BMDM was much more heterogeneous, with a higher % of less responsive cells. The results indicate that the TAK1[D175A] BMDM do not consist of 90% of cells in which WT TAK1 has been completely replaced by TAK1[D175A] and 10% of cells containing WT TAK1, but instead consist of a population of BMDM with variable levels of residual WT TAK1 (Supplementary Figure S4C).

### Role of TAK1 in initiating signal transduction in mouse BMDM

**Figure 5.**
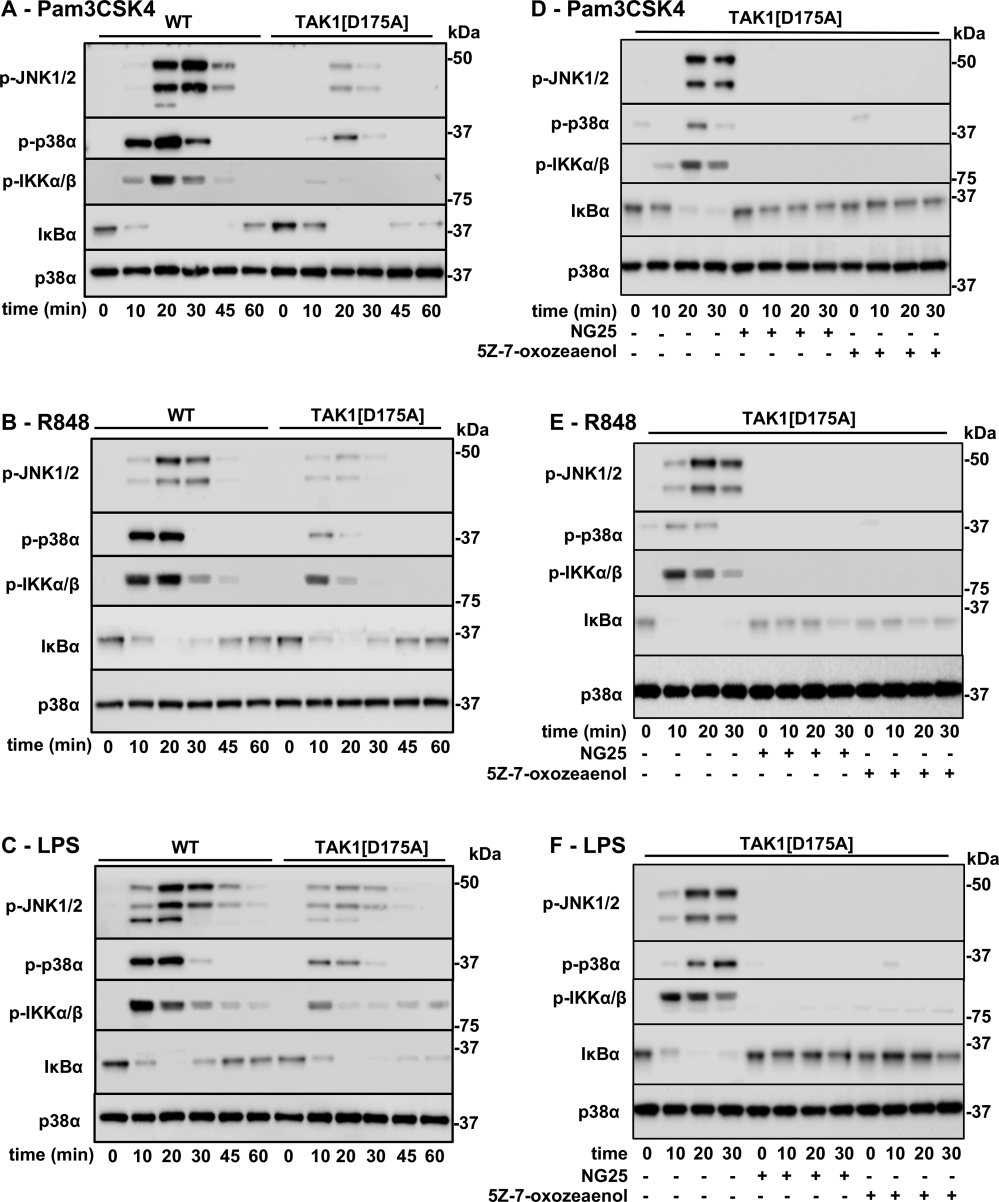
TAK1 catalytic activity is required for Pam_3_CSK_4_-, R848- and LPS-stimulated signalling in BMDM. (**A**–**C**) BMDM from WT and/or TAK1[D175A] mice were stimulated for the times indicated with 1 µg/ml Pam_3_CSK_4_ (**A**), 250 ng/ml R848 (**B**) or 100 ng/ml LPS (**C**). The cell extracts (10 µg protein) were subjected to SDS–PAGE and immunoblotted with antibodies recognising phosphorylated (p) forms of JNK1/2, p38α, IKKα/β, and for all forms of IκBα and p38α MAPK (p38α). Similar results were obtained in 3 separate experiments. (**D**–**F**) as in **A**–**C**, except that BMDM from TAK1[D175A] mice were incubated for 1 h without (−) or with (+) the TAK1 inhibitors NG25 (2 µM) or 5Z-7-oxozeaenol (3 µM).

We found that the Pam3CSK4, R848 or LPS-stimulated phosphorylation (activation) of JNK1/2, p38α MAP kinase (p38α) and IκB kinases (IKKs) peaked after 20 min in macrophages and declined thereafter. The activation of these protein kinases was reduced, but not abolished in BMDM from TAK1[D175A] × Vav-iCre mice (Figure 5A–C). The partial reduction in TLR signalling could be explained in two ways. First, the residual signalling might be catalysed by another protein kinase, distinct from TAK1; second it might be catalysed by the small amount of residual WT TAK1 activity still present in the BMDM preparations from TAK1[D175A] × Vav-iCre mice. To distinguish between these possibilities, we studied the effects of two potent but structurally unrelated inhibitors of TAK1, 5Z-7-oxozeaenol [22] and NG25 [23] (Supplementary Figure S5A). 5Z-7-oxozeaenol is a covalent inhibitor of TAK1 [22] while NG25 is a Type II protein kinase inhibitor.

We first studied the specificities of these compounds towards many other protein kinases, including most members of the MAP3K, MAP4K, STE20 and PAK kinases to which TAK1 is most closely related (Table 1). These experiments revealed 5Z-7-oxozeaenol is not only a potent inhibitor of TAK1 (MAP3K7), but also a potent inhibitor of other MAP3K family members (ZAK, ARAF, BRAF and RAF1) and a less potent inhibitor of the STE20 family members LOK and SLK. In contrast, the TAK1 inhibitor NG25 does not inhibit any MAP3K or MAPK4 family members apart from ZAK (MAP3K20) and GCK (MAP4K2) and does not inhibit LOK or SLK. The specificities of 5Z-7-oxozeaenol and NG25 have also been profiled against over 100 other protein kinases by the MRC-PPU international kinase profiling centre (https://www.kinase-screen.mrc.ac.uk/inhibitor-and-results/277 and https://www.kinase-screen.mrc.ac.uk/inhibitor-and-results/177) and revealed no overlap in the off-target effects of these inhibitors. NG25 inhibited ABL, p38α MAP kinase and Src family members (LCK, YES and SRC) and (less potently) OSR1, JNK2, CAMK1, CSK, MINK1 and members of the EPHRIN kinase subfamily. In contrast, 5Z-7-oxozeaenol inhibited ERK8 and MEKs 1, 2 and 6 and (less strongly) BRK, PRAK, TRKA and VEGFR1. The only protein kinase that was inhibited potently by both NG25 and 5Z-7-oxozeaenol was therefore ZAK (MAP3K20) (Table 1). However, ZAK is expressed most highly in skeletal muscle and cardiac muscle and patients with homozygous ZAK mutations [24] or ZAK-deficient mice [25] display muscle weakness and immune defects have not been reported. The experiments indicated that NG25 and 5Z-7-oxozeaenol could be used together to probe the identity of the ‘upstream' protein kinase(s) mediating the residual activation of the IKK complex and MAP kinases in BMDM from TAK1[D175A] mice.

**Table 1:** Specificity of 5Z-7-oxozeanol and NG25 towards the MAP3K, MAP4K and STE20 families of protein kinases

Protein kinase	Alternative name	% activity remaining	
		5Z-7-Oxozeanol	NG25
MAP3K2	MEKK2	98	92
MAP3K3	MEKK3	111	42
MAP3K5	ASK1	97	112
MAP3K7	TAK1	−1	3
MAP3K9	MLK1	77	119
MAP3K10	MLK2	102	101
MAP3K16	TAO1	67	67
MAP3K17	TAO2	91	50
MAP3K18	TAO3	86	51
MAP3K20	ZAK	−3	−1
ARAF	no other name	2	95
BRAF^1^	no other name	2	108
RAF1	c-RAF	1	120
MAP4K1	HPK1	104	11
MAP4K2	GCK	81	4
MAP4K3	GLK	96	87
MAP4K4	HGK	95	141
MAP4K5	GCKR	68	32
MAP4K6	MINK	111	89
MST1	STK4	97	110
MST2	STK3	95	100
MST3	STK24	68	127
MST4	STK26	109	106
PAK1	PAKa	113	113
PAK2	PAKg	105	98
PAK3	bPAK	88	101
PAK4	no other name	90	99
PAK5	no other name	110	111
PAK6	no other name	98	92
LOK	STK10	27	90
SLK	STK2	20	115
TNIK	no other name	70	141
GCN2	no other name	90	100
STK25	YSK1	98	107
MYO3B	no other name	99	104
WNK2	no other name	104	106
WNK3	no other name	102	99

The assays were performed at Eurofins, according to their published protocols. Each compound was used at a final concentration of 1 µM and the ATP concentration in the assay was 0.1 mM. All the assays were repeated a second time with similar results. The results are presented as % activity remaining in the presence of inhibitor relative to the activity measured in the absence of inhibitor (100%). A negative number indicates that the value obtained was marginally lower than that measured in assay blanks where both inhibitor and kinase were omitted.

1 The oncogenic BRAF[Val600Glu] mutant was used in the assays.

The residual Pam3CSK4-, R848- or LPS-dependent activation of JNK1/2, p38α and IKKα/β in BMDM from the TAK1[D175A] × Vav-iCre knock-in mice was suppressed by either 5Z-7-oxozeaenol or NG25 (Figure 5D–F), indicating that the residual signalling observed in BMDM from TAK1[D175A] × Vav-iCre mice is catalysed by the small amounts of WT TAK1 present in these cells. The TAK1 inhibitors did not cause significant dissociation of the TAK1 catalytic subunit from its regulatory subunits TAB1, TAB2 and TAB3 (Supplementary Figure S5B) and these components also remained complexed to the kinase inactive TAK1[D175A] mutant in BMDM from the TAK1[D175A] knock-in mice (Supplementary Figure S5C).

### TAK1 is required for TLR signalling in human THP1 cells

To investigate whether TAK1 catalytic activity was also important for TLR-dependent signalling and cytokine production in human myeloid cells, we performed further studies using the human monocyte-derived cell line THP1, which can be differentiated to a macrophage-like phenotype with phorbol myristate acetate (PMA). We made TAK1 knock-out (KO) monocytes using CRISPR/Cas9 gene-editing technology and two independently generated TAK1 KO clonal cell lines (C4 and C5) were used for these studies. In TAK1 KO cells the expression of the regulatory subunits of the TAK1 complex, TAB1, TAB2 and TAB3, was comparable to the parent THP1 cells from which they were derived (Supplementary Figure S6A) and the expression of the protein components of the Myddosome, MyD88 and IRAKs 1-4 were also little affected (Supplementary Figure S6B).

**Figure 6.**
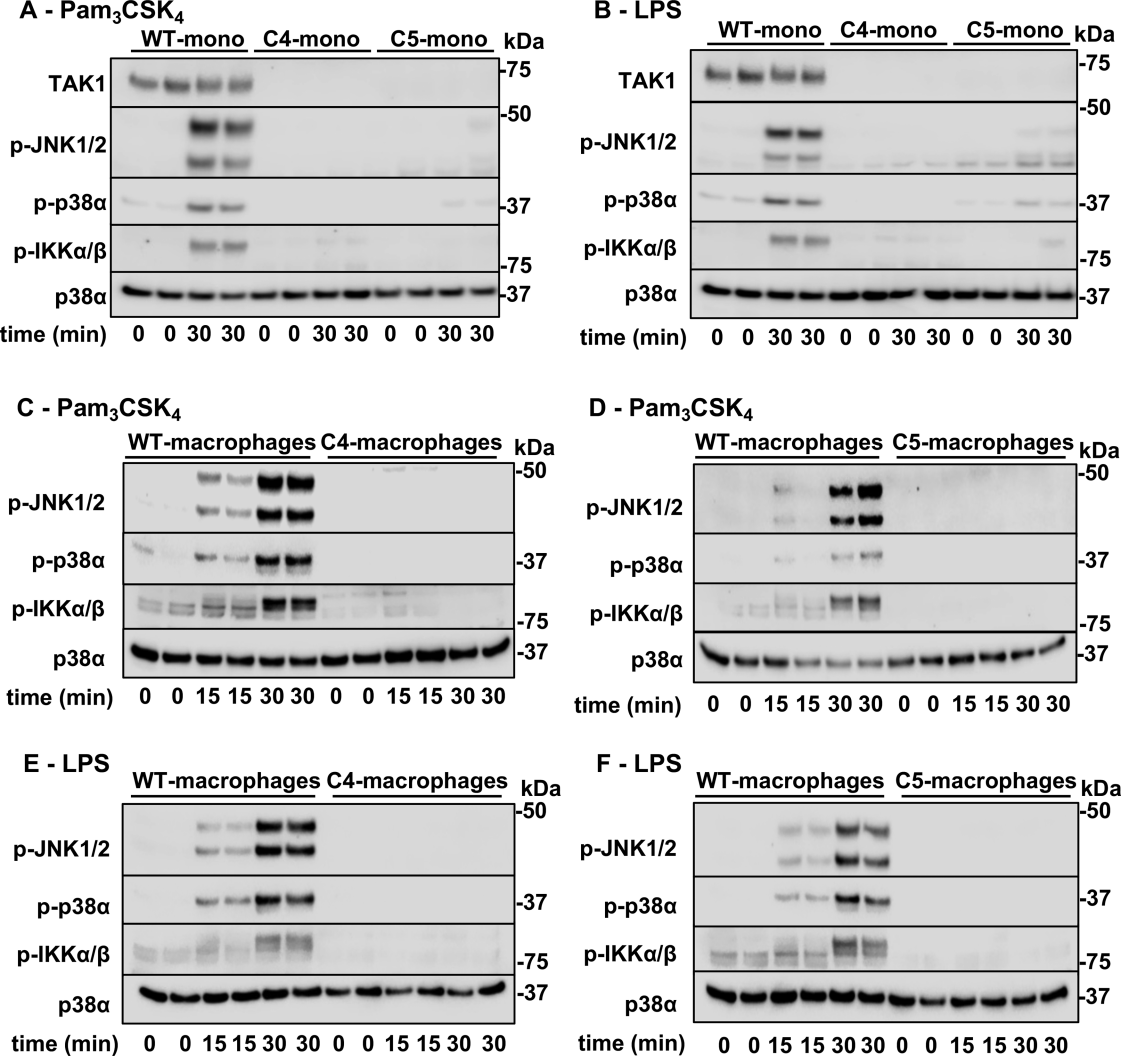
TAK1 is required for Pam3CSK4- and LPS- stimulated signalling in THP-1 monocytes and THP-1 macrophages. WT or TAK1 KO THP1 monocytes clone 4 and clone 5 (C4-Mono and C5-Mono) were stimulated for 30 min with 100 ng/ml Pam_3_CSK_4_ (**A**) or 100 ng/ml LPS (**B**). (**C**–**F**) WT and TAK1 KO THP-1 cells were differentiated to a macrophage-like phenotype by stimulation with PMA (see Methods) then stimulated with Pam3CSK4 (**C**, **D**) or LPS (**E**, **F**) as in A. (**A**–**F**) SDS-denatured cell extracts (10 µg protein) were subjected to SDS–PAGE and immunoblotting with antibodies recognising human TAK1, the phosphorylated (p) forms of JNK1/2, p38α MAPK (p38α), IKKα/β and all forms of p38α (p38α). Similar results were obtained in three separate experiments.

**Figure 7.**
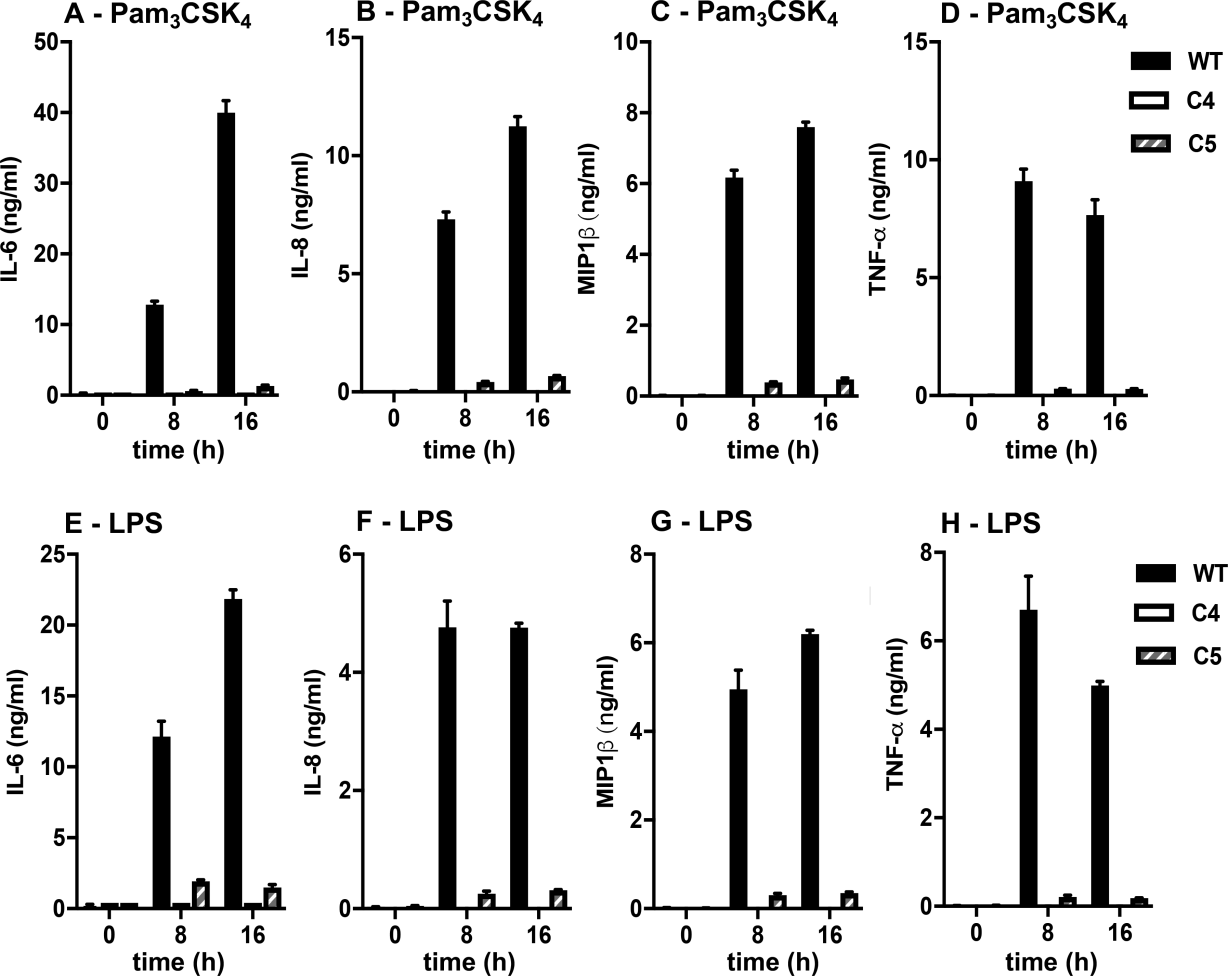
TAK1 is required for Pam_3_CSK_4_- and LPS-stimulated cytokine secretion in THP-1 monocytes. WT THP-1 cells (black bars), TAK1 KO THP1 cells clone 4 (white bars-levels are baseline) and TAK1 KO THP1 cells clone 5 (hatched bars) were stimulated for 8 h or 16 h with 100 ng/ml Pam_3_CSK_4_ (**A**–**D**) or 100 ng/ml LPS (**E**–**H**) and the secretion of IL-6 (**A**, **E**), IL-8 (**B**, **F**), macrophage inflammatory protein (MIP)-1β (**C**, **G**) and TNF (**D**, **H**) in the culture medium was measured. Experiments were performed with duplicate dishes of cells and the graphs show the average values. Similar results were obtained in three separate experiments.

THP1 cells are responsive to Pam3CSK4 and LPS, but not R848. We found that the Pam3CSK4 or LPS-stimulated activation of JNK1/2, p38α and IKKα/β was abolished in TAK1 KO THP1 monocytes (Figure 6A,B) and similar results were obtained when the monocytes were differentiated to macrophages (Figure 6C–F). Consistent with these observations, the Pam3CSK4- or LPS-stimulated secretion of IL-6, IL-8, macrophage inflammatory protein (MIP)-1β and TNF in THP1 monocytes were reduced drastically (Figure 7A–G).

## Discussion

The studies reported in this paper establish that the catalytic activity of TAK1 is required to initiate signalling in myeloid cells by TLRs that signal via MyD88. Protein kinase cascades have considerable amplification potential and relatively little activation of the first kinase in the cascade may be needed to elicit near maximal phosphorylation of substrates of the most downstream kinase in the cascade [26]. This phenomenon was observed in BMDM from TAK1[D175A] × Vav-iCre mice in the present study, in which the small amount of residual wild-type TAK1 activity present in these cells was sufficient to induce significant activation of IKKβ which, in turn, was sufficient to induce a similar rate of degradation of IκBα (triggered by the IKKβ-catalysed phosphorylation of IκBα) to that observed in BMDM from WT mice (Figure 5A–C). Only when the residual wild-type TAK1 was suppressed by the TAK1 inhibitors 5Z-7-oxozeaenol or NG25, was the degradation of IκBα prevented (Figure 5D–F).

Consistent with the requirement of TAK1 catalytic activity for MyD88-dependent signalling, we found that the secretion of IL-6, IL-10, IL-12 and TNF induced by Pam3CSK4 or R848 was strongly suppressed in BMDM and neutrophils from TAK1[D175A] × Vav-iCre mice at all time points examined (Figure 2) and this was also true for LPS signalling in neutrophils (Figure 4). In contrast, IL-6 and TNF secretion induced by LPS in BMDM from TAK1[D175A] × Vav-iCre mice was only reduced up to 4 h of stimulation and then increased so that no difference in total IL-6 and TNF secretion was observed after 8 h. In contrast with other TLRs, TLR4 signals via TRIF as well as MyD88 in BMDM, and it is well established that in these cells TRIF-dependent signalling becomes rate limiting for cytokine production after prolonged stimulation with LPS [4]. Our results suggest that TAK1 catalytic activity may not be required for the TRIF dependent production of IL-6 and TNF in BMDM, although it is required for the LPS-stimulated production of IL-10 and IL-12 (Figure 2I–L). Alternatively, or in addition, the increased production of the pro-inflammatory cytokines IL-6 and TNF might be caused indirectly by reduced production of the anti-inflammatory cytokine IL-10, which is known to suppress production of pro-inflammatory cytokines. It is also possible that type-1 interferons, which are produced by the TRIF-dependent but not by the MyD88-dependent pathway, affect the LPS-stimulated formation and secretion of IL-6 and TNF in BMDM from TAK1[D175A] × Vav-iCre mice.

The reason why the LPS-stimulated secretion of IL-6 and TNF is strongly suppressed in TAK1[D175A] neutrophils (Figure 4G,I) but not in BMDM (Figure 2I,L) is unclear. However, one interesting possibility is that TLR4 does not translocate from the plasma membrane to endosomes in these cells so that LPS is unable to signal via TRIF from endosomes. Thus, LPS dependent signalling in neutrophils may be largely mediated via Myddosomes. Another possibility is that the replacement of WT TAK1 by the kinase-inactive TAK1[D175A] mutant is essentially 100% complete in neutrophils in contrast with BMDM.

Although we found that the LPS-stimulated secretion of IL-6, IL-12p40 and TNF was strongly reduced in peritoneal neutrophils, other investigators reported previously that the LPS-stimulated production of IL-6 and TNF was enhanced in peritoneal neutrophils from TAK1 KO × LyzM-Cre mice[19] or TAK1[Δ40–78] × LyzM-Cre mice. The reasons for this striking difference could be explained in a number of ways. First, the reduced expression of the TAK1 protein may enable another protein kinase, perhaps another MAP3K family member, such as MEKK3 [27], to replace TAK1 in the TLR signalling pathway. In this abnormal situation, the feedback control mechanisms that prevent the hyperactivation of TAK1 [11,28] may not operate, leading to deregulated TLR signalling and the over-production of pro-inflammatory cytokines. Second, in myeloid cells from TAK1 KO × LyzM-Cre mice expressing reduced levels of the TAK1 protein, the regulatory subunits TAB1, TAB2 and TAB3 (see introduction) may still be expressed at normal levels but not bound to TAK1. This abnormal situation might also have unanticipated effects on signalling and cytokine production that are unrelated to the loss of TAK1 activity. Third, increased apoptosis in myeloid cells from TAK1 KO × LyzM-Cre mice might trigger secondary inflammations that drive the enhanced production of IL-6 and TNF by TAK1-independent pathways [29]. This indirect mechanism might also contribute to the enhanced production of IL-6 and TNF that we observed between 4 and 8h of stimulation with LPS in BMDM from TAK1[D175A] × Vav-iCre mice.

## Materials and methods

### Generation of a conditional D175A knock-in mutation in the *Map3k7* gene encoding TAK1

The TAK1[D175A] mice were produced by Taconic-Artemis using a targeting vector designed by JSCA. To make this mouse, a minigene strategy was used to express wild-type TAK1 in the absence of Cre recombinase. A targeting vector was therefore constructed, which upon expression of Cre recombinase should excise the mini-gene and allow the TAK1[D175A] mutant to be expressed (Supplementary Figure S1A). In addition to 5′ and 3′ arms of homology, the targeting vector also contained a loxP flanked cassette consisting of a neomycin resistance gene flanked by FRT sites, the 3′ end of exon 5 and the splice acceptor site for exon 6 joined to a minigene consisting of exons 6 to 17 of the *Map3k7* gene, followed by a human growth hormone polyadenylation (hGHpA) sequence and finally an F3-flanked puromycin resistance gene. The D175A mutation was introduced in exon 6 at the start of the 3′ arm of homology. The vector was linearised using Not 1 and transfected into Art B6 3.6 ES cells, which were generated from a C57BL/6 N Tac genetic background. After selection with G418 and puromycin, colonies were expanded and tested for the correct homologous recombination at the *Map3k7* gene locus. Positive clones were confirmed by Southern blotting with 5′ and 3′ probes external to the targeting vector (Supplementary Figure S1B) as well as internal probes to confirm integration of the targeting vector. The presence of the D175A mutation in exon 6 was confirmed by sequencing of PCR products in this region (data not shown). Chimeric mice from correctly targeted ES clones were generated by blastocyst injection. Male chimeric mice were crossed to Flpe transgenic (C57BL/6-*Tg^(CAG-Flpe)2Arte)^* mice, resulting in the removal of the neomycin and puromycin resistance genes in offspring with germline transmission of the mutated *Map3k7* allele. Heterozygous mice with the conditional *Map3k7* allele were backcrossed with C57Bl6/J mice for three generations. From the first generation of backcrossing, mice negative for the Flpe transgene were selected for further backcrossing. To generate the mice with a D175A knock-in in the Map3k7 allele, mice were crossed onto a Vav-iCre transgene [30]. Due to potential recombination in the male germline with this Cre transgene, only female Cre positive mice were used for further breeding. Routine genotyping was carried out by PCR of ear biopsies using the following primers *Map3k7* Forward 5′CATTGGTCTGCAGCTATAGCTCAG3′, *Map3k7* Reverse 5′CATTGGTCTGCAGCTATAGCTCAG3′, *Vav-Cre* Forward: 5′CTCCAACCTGCTGACTGTGC3′ and *Vav-Cre* Reverse 5′CACCAGGGACACAGCATTGG 3′. *Map3k7*^flox/flox^ mice were used as WT controls and *Map3k7*^flox/flox^Vav^iCre/+^ mice are referred as TAK1[D175A] × Vav-iCre.

Wild-type C57Bl6J mice for backcrossing were obtained from Charles River Laboratories UK. Animals were maintained in the Resource Unit at the University of Dundee in individually ventilated cages under specific pathogen free conditions and housed in line with UK and EU regulations. Nonbreeding mice were kept in same sex groups and allowed free access to water and food (R&M3 SDS, Special Diet Services). Animal rooms were maintained on a 12/12h light/dark cycle at 21°C between 45 and 65% humidity. Mice were killed using a rising concentration of carbon dioxide (CO_2_) in their home cage and death was confirmed by cervical dislocation. All work was carried out under a UK Home Office licence (PAAE38C7B) and approved by the University of Dundee Ethical review and Welfare Committee.

### Flow cytometry

Spleen and lymph node cell suspensions were obtained as described previously [31]. For flow cytometry analysis, the cells were blocked for 20 min at 4°C with FcR antibody (purified anti-CD16/32; BD Pharmigen) diluted (1 : 50) in PBS containing 1% Bovine serum albumin. For detection of surface antigens, the cells were stained for 20 min at 4°C with the appropriate fluorophore-conjugated antibodies from BioLegend (anti-CD11b PE/Cy7 (catalogue number #101216, 1:600 dilution), anti-Ly6G APC (#127613, 1 : 400 dilution), anti-Ly6C FITC (#128005, 1 : 400 dilution), anti-F4/80 PE (#123110 1 : 200 dilution), anti-TCRb APC/Cy7 (#109220 1 : 50 dilution), anti-CD19 APC (#115512, 1 : 200 dilution), anti-CD8 PE/Cy7 (#100722, 1 : 200 dilution), anti-CD44 FITC (#103022,1 : 200 dilution), anti-CD62L PE (#104407, 1 : 200 dilution), anti-CD4 PerCP/Cy5.5 (#100434, 1 : 200 dilution). To measure apoptosis, BMDM were stained with APC Annexin V Apoptosis Detection Kit using propidium iodide (#640932 from BioLegend) according to the manufacturer's instructions.

### Generation of bone marrow-derived macrophages (BMDM) and isolation of peritoneal neutrophils

BMDM were generated by differentiating bone marrow obtained from the femur and tibia using L929 preconditioned medium, as described [4]. On day 7 adherent BMDM were re-plated in fresh culture medium in 24-well (0.25 × 10^6^ cells in 500 µl media) or 6-well (1 × 10^6^ cells in 1 ml media) tissue culture plates. Cells were allowed to adhere for 16 h and the following day were stimulated with TLR ligands at concentrations and time points specified in figure legends.

Peritoneal neutrophils were isolated from casein injected mice. To recruit neutrophils to the peritoneum, a 9% (w/v) solution of casein was injected intraperitoneally (20 ml/kg). After 16 h a second injection of casein was made and the mice killed after a further 3 h. The peritoneal exudates were collected by injecting 3 ml of filter-sterilised PBS (Phosphate Buffered Saline) containing 0.02% (w/v) EthyleneDiamineTetracetic Acid (EDTA). The cells were washed three times with PBS and purified with EasySep™ Mouse Neutrophil Enrichment Kit (#19762, Stem Cell Technologies) according to the manufacturer's recommendations. The purity of all neutrophil preparations was at least 86%. Cells were stained with 0.5 µg/ml DAPI (4′,6-diamidino-2-phenylindole) (#422801, BioLegend) and counted using a NovoCyte flow cytometer. Cells were then suspended in Roswell Park Memorial Institute Medium (RPMI) medium with 10% (v/v) FBS and plated in 96 (0.05 × 10^6^ cells in 100 µl media) or 24-well plates (0.2 × 10^6^ cells in 400 µl media), before stimulating with TLR ligands at the concentrations and time points specified in figure legends.

### Culture of human THP-1 cells and generation of TAK1 knockout THP-1 cells

THP-1 cells (#TIB-202™, ATCC) were grown in suspension in a humidified 37°C, 5% CO_2_ incubator in Iscove's Modified Dulbecco's Medium (IMDM, Life Technologies) supplemented with 2 mM glutamine, 10% (v/v) fetal bovine serum (FBS), and the antibiotics streptomycin (0.1 mg/ml) and penicillin (100 U/ml). Wild type (WT) and TAK1 KO THP-1 monocytes were differentiated into macrophages by culturing for 72 h in presence of 200 ng/ml PMA, washed twice with PBS to remove traces of PMA and resuspended in normal IMDM growth medium for a further 24 h prior to stimulation.

The guide RNAs used to target Exon 1 of the human TAK1 gene were synthesised by the DNA cloning team in the MRC-PPU. The guide RNAs used were:- GAGGGGCTTCGATCATCTCAC (DU64616) and GCAAGGAGATCGAGGTGGAAG (DU64617) and are available from https://mrcppureagents.dundee.ac.uk/ on request. The TAK1 KO THP-1 cells were generated using lentiviral transduction. To generate lentiviral particles, 3.25 µg of lentiviral vector (lentiCRISPR v2 (Plasmid #52961, Addgene), 2 µg of vector encoding gag/pol packaging protein and 1.25 µg of vector encoding vesicular stomatitis virus G protein (VSV-G) envelope proteins were diluted in Opti-MEM reduced serum media (Gibco). The components were mixed with 25 µl Lipofectamine 2000 (Invitrogen) diluted in Opti-MEM reduced serum media and incubated for 15 min at RT. The solution was added drop-wise to 293FT (#R70007, Thermofisher) cells cultured in 10 cm dishes at 90% confluency in Opti-MEM reduced serum media. The culture medium was replaced with fresh media 4 h after transfection and, after a further 48 h, the media containing lentivirus was collected and passed through a 0.45 µm filter to remove cell debris. 0.5 ml lentivirus-containing media was added to 500 000 THP-1 cells with 2 µg/ml polybrene to facilitate infection. The media was refreshed 24 h after virus infection and, 24 h later, cells were selected for 1–2 weeks in media containing 1 μg/ml puromycin. The cells were then single-cell plated into 96-well plates and left until colonies began to form (3–4 weeks). The mutational efficiency was analyzed by immunoblotting the cell extracts for TAK1. Multiple TAK1 knockout (KO) clones were obtained, a few of which were selected for further study.

### Preparation of cell extracts

Cells were washed with ice cold PBS and lysed in 50 mM Tris–HCl, pH 7.4, 1 mM EDTA, 1 mM EGTA (Ethylene glycol-bis(2-aminoethylether)-N,N,N′,N′-tetraacetic acid), 50 mM sodium fluoride, 5 mM sodium pyrophosphate, 10 mM sodium 2-glycerol 1-phosphate, 1 mM sodium orthovanadate, 0.27 M sucrose, 1% (v/v) Triton X-100, 1 μg/ml aprotinin, 1 μg/ml leupeptin, 1 mM phenylmethylsulphonyl fluoride and 1.0 mM dithiothreitol, centrifuged at 4°C for 15 min at 16 000×***g*** the supernatants, termed cell extracts, were collected and their protein concentrations determined by the Bradford assay.

### Cytokine secretion

Following stimulation of mouse BMDM, peritoneal neutrophils or human THP1 cells, the cell culture medium was collected, clarified by centrifugation for 10 min at 14 000×***g*** and stored at −80°C prior to analysis. Cytokine concentrations in the supernatants were measured using Luminex-based Bio-Plex assays for human or mouse cytokines (Bio-Rad) according to the manufacturer's instructions. The standard cytokines used for calibration were reconstituted in fresh culture medium. Plates were read using a Luminex 100/200 machine and analysed using xPONENT® software.

### Quantitative real-time PCR

To investigate the proportion of WT and TAK1[D175A] mutant present in blood leukocytes or BMDM from TAK1[D175A] mice, RNA was isolated from these cells using the Mouse RiboPure™-Blood RNA Isolation Kit (#AM1951, ThermoFisher) or the EZNA microelute total RNA kit (#R683101, VWR) and reverse transcribed into cDNA using an iScript™ cDNA Synthesis Kit (#170-8891, Bio-Rad). The qPCR reaction was set up using PrimeTime® Gene Expression Master Mix (#1055770, Integrated DNA Technologies), *Map3k7* forward (5′-TGATTCACAGGGACCTCAAG-3′) and *Map3k7* reverse (5′-TGTGTGTTTGGATGTCACAAG-3′) primers were used at 400 nM. Custom-made PrimeTime LNA WT TAK1 probe (5′-TCTGCGATTTTGGT-3′) and mutant TAK1 probe (5′-CTGCGCATTTGGT-3′) consisted of a FAM™ or Yakima Yellow^R^ reporter dye at the 5′ end of each probe and Iowa Black FQ quencher at the 3′ end. Each probe was used at 200 nM. Primers and probes were purchased from Integrated DNA Technologies. The percentage of TAK1 mutant cDNA sequence was calculated according to the equation:

$$\text{\%}\mathrm{Seq}1=100\times (2^{\mathrm{Seq}2\mathrm{Ct}-\mathrm{Seq}1\mathrm{Ct}})/(1+2^{\mathrm{Seq}2\mathrm{Ct}-\mathrm{Seq}1\mathrm{Ct}}),$$

where Seq1 is the mutant sequence and Seq2 is the WT sequence [32]. The qPCR was carried out in a CFX384 PCR Detection System (Bio-Rad), with a 3 min activation step at 95°C followed by 45 cycles of amplification (15 s at 95°C and 60 s at 60°C). Data were analysed using CFX Manager™ Software.

To measure cytokine RNAs, qPCR was carried out using SsoFast™ EvaGreen Supermix (Bio-Rad) and primers for measuring *il6 (*forward primer: 5′-TTCCATCCAGTTGCCTTCTTG-3′ and reverse primer: 5′-AGGTCTGTTGGGAGTGGTATC-3′), *il10* (forward primer: 5′-CCCTTTGCTATGGTGTCCTTTC-3′ and reverse primer: 5′-GATCTCCCTGGTTTCTCTTCCC-3′) and *il12p40* mRNA (forward primer: 5′-TCATCAGGGACATCATCAAACC-3′ and 5′-TGAGGGAGAAGTAGGAATGGG-3′). Values were normalised to *gapdh* mRNA (forward primer: 5′-TGCACCACCAACTGCTTAG-3′ and reverse primer: 5′-GATGCAGGGATGATGTTC-3′) and fold-induction calculated relative to the cytokine RNA levels measured in unstimulated wild-type cells [33].

### Antibodies and other reagents

Antibodies recognising JNK1 and JNK2 phosphorylated at Thr183 and Tyr185 (#9251), p38α MAPK phosphorylated at Thr180 and Tyr182 (#9211), IKKα phosphorylated at Ser176 and Ser180 and IKKβ phosphorylated at Ser177 and Ser181 (#2697). The phosphorylation of these sites is known to be essential for the activation of JNK1/2, p38α MAPK and IKKα/β. These antibodies and those recognising all forms of TAK1 (#4505), IκBα (#(L35A5), TAB2 (#3745), TAB3 (#14241), IRAK1 (#4504), IRAK2 (#4367), IRAK3 (#4369), IRAK4 (#4363), MyD88 (#4823), p38α MAP kinase (#9212) and GAPDH (#2118) were purchased from Cell Signaling Technology and used at 1 : 1000 dilution. An antibody recognising TAB1 (#ab151408) was obtained from Abcam. Antibodies recognising TAK1 that were made in sheep (#S527, KKQLEVIRSQQQKRQGTS [residues 562–579 of human]) and preimmune control sheep IgG were purchased from MRC-PPU reagents and services. Secondary anti-rabbit (#7074S) and anti-mouse (#7076S) antibodies coupled to horseradish peroxidase were obtained from Cell Signalling Technology and used at a 1 : 5000 dilution. Pam3CSK4 (tlrl-pms) was purchased from Invivogen, LPS (*Escherichia coli* strain O5:B55) from Enzo Life Sciences (#ALX-581-013-L002) and Phorbol 12-Myristate 13-Acetate (PMA) from Sigma–Aldrich. The TAK1 inhibitor NG-25 was synthesised by Dr Natalia Shpiro, MRC Protein Phosphorylation and Ubiquitylation Unit, University of Dundee, and 5Z-7-oxozeaenol was purchased from Tocris (#3604).

### Statistical analysis

All the experiments were repeated at least four times with similar results. Statistical analyses were performed with GraphPad Prism Software, and quantitative data in graphs and bar charts are presented as the arithmetic mean ± SEM. Statistical significance of differences between experimental groups was assessed in graphs and bar charts, using the student *t*-test or Mann–Whitney test.

## Data Availability

The authors confirm that all relevant data has been provided in the main article or as Supplementary Material.

## Abbreviations

BMDM: bone marrow-derived macrophages
GAPDH: glyceraldehyde 3-phosphate dehydrogenase
HEK: human embryonic kidney
IKK: IκB kinase
IL-1: interleukin-1
IRAK: interleukin-1 receptor (IL-1R) associated kinase
JNK: c-jun N-terminal kinase
LPS: lipopolysaccharide
MAPK: mitogen-activated protein kinase
MEKK: MAPK or ERK kinase
MKK: MAPK kinase
MyD88: myeloid differentiation primary response 88
PAMP: pathogen associated molecular pattern
PMA: phorbol myristate acetate
TAB: TAK1-binding
TAK: TGFβ-activated kinase
TLR: toll-like receptor
TRAF6: TNF-Receptor-Associated Factor 6
TRIF: TIR-domain-containing adapter-inducing interferon-β
