# Supplementary material for: TAK1 protein kinase activity is required for TLR signalling and cytokine production in myeloid cells
Source: Biochem J. 2022 Sep 16;479(17):1891–907. doi: 10.1042/BCJ20220314 (PMC9555797; doi:10.1042/BCJ20220314)
Supplement: Supplementary Material [file BCJ-479-1891-s1.pdf]

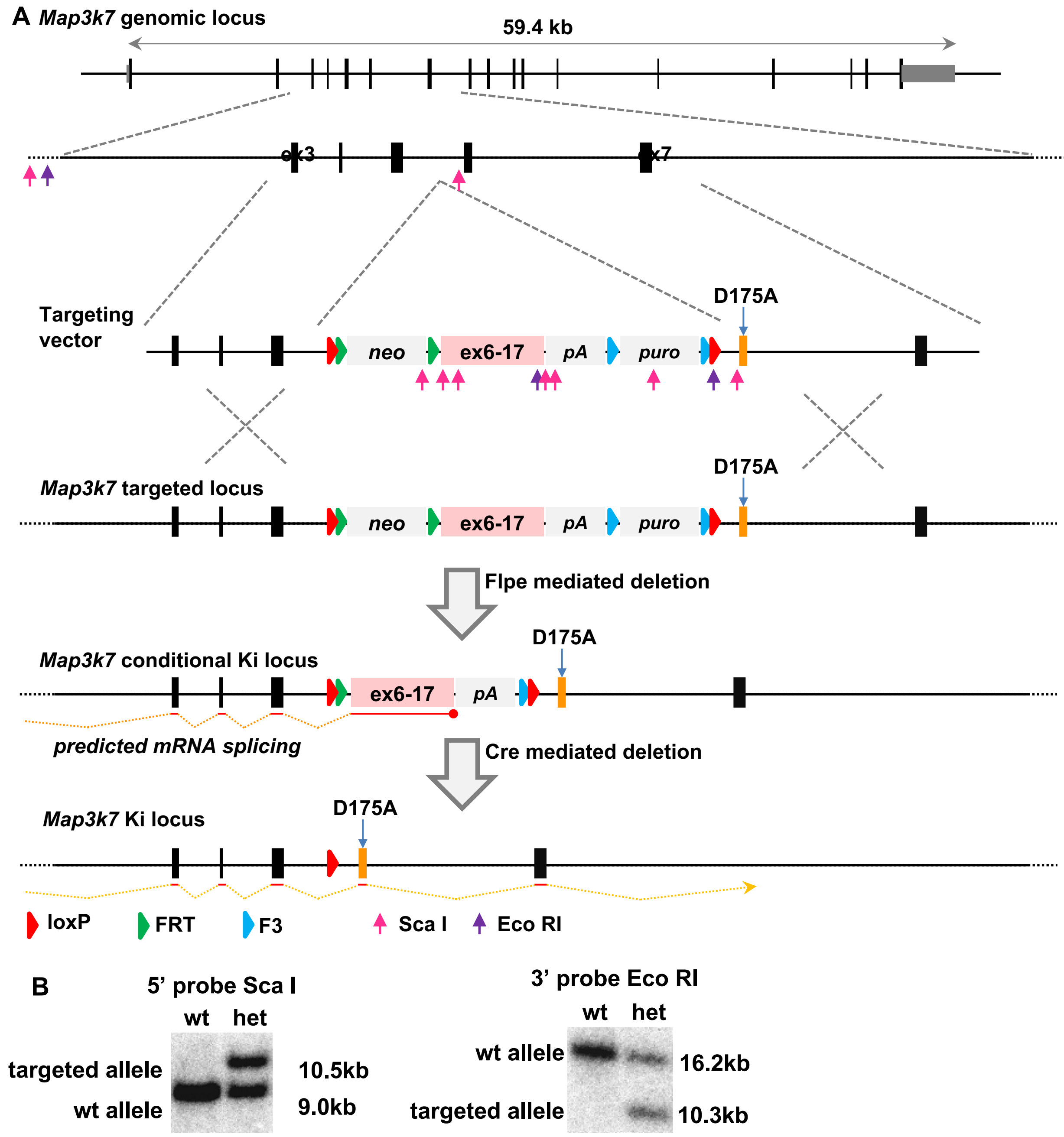

Supplementary Figure S1. Generation of a conditional D175A mutation in the *map3k7* gene encoding TAK1.

Asp175 of TAK1 is encoded within exon 6 of the Map3k7 gene. The strategy used to create a conditional D175A knock-in mutation is shown in (**A**). A targeting vector was used to insert a cassette containing a neomycin resistance (neo) gene flanked by FRT sites, the 3' end of exon 5 and the splice acceptor site for exon 6, joined to a minigene consisting of exon 6 to 17 of the Map3k7 gene, followed by an hGHpA sequence and then a F3-flanked puromycin resistance (puro) gene. This cassette was flanked by LoxP sequences. Following transfection of the targeting vector into ES cells, correctly targeted clones were identified by a combination of Southern blotting using probes external to the targeting vector (**B**) and PCR analysis (not shown). ES cells were used to generate chimeric mice, which were crossed to Flpe transgenic mice. This resulted in the deletion of the neomycin and puromycin genes. In mice with the conditional Map3k7 allele, transcription of the Map3k7 gene would be driven via the endogenous Map3k7 promoter but splicing from exon2 onto the minigene would result in a mRNA encoding wild type TAK1. Following expression of Cre, the minigene would be removed, resulting in mRNA encoding the TAK1[D175A] mutation.

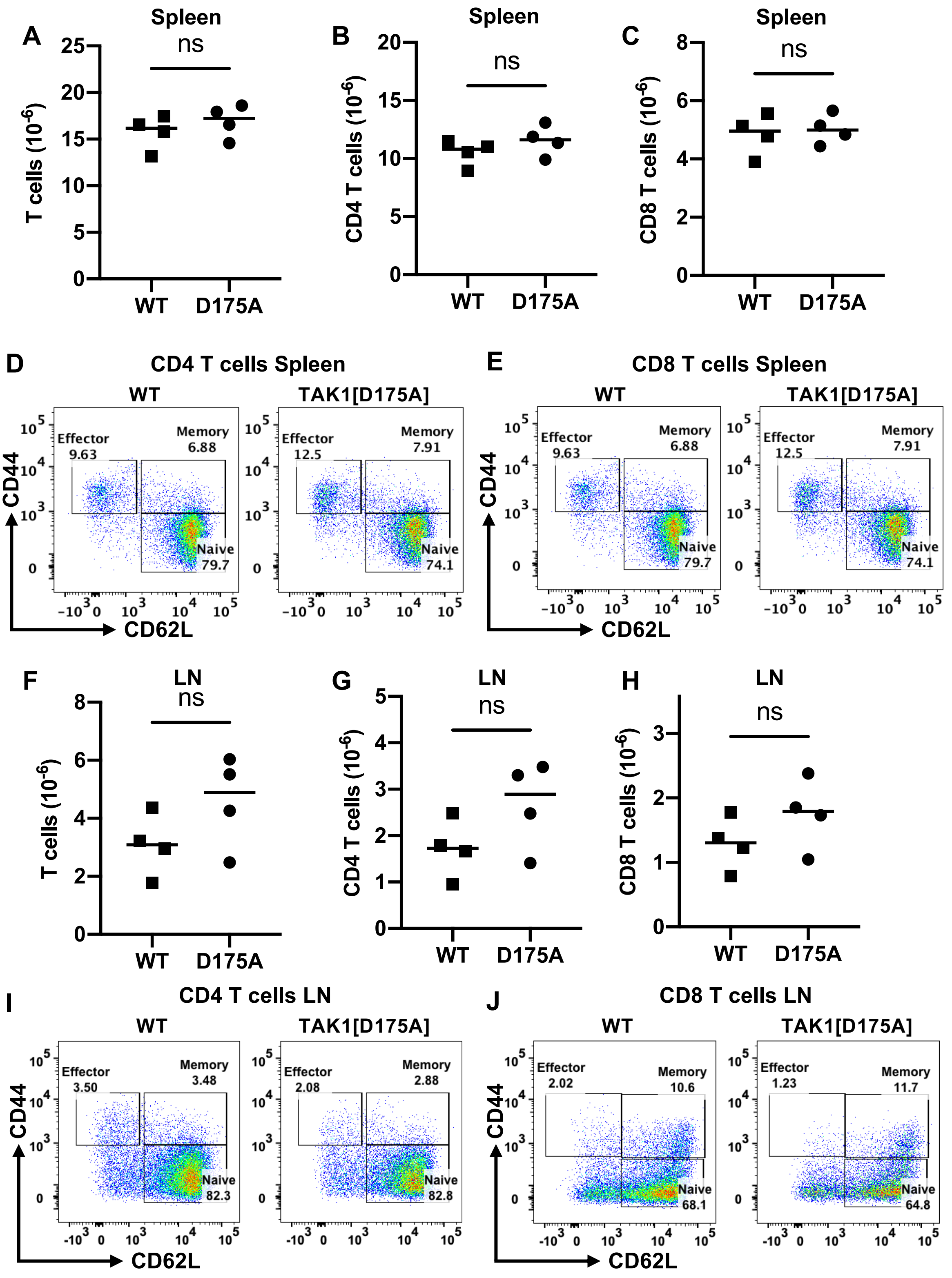

Supplementary Figure S2. T cell numbers and their activation state are normal in TAK1[S175A] x Vav-iCre mice.

(**A**) T cell (DAPI$^-$TCRβ$^+$) numbers in the spleen of 12 week old WT (n=4) and TAK1[D175A] x Vav-iCre mice (n=4) determined by flow cytometry. Each symbol represents an individual mouse. (**B-C**) As in (A), except plots show CD4 T cell (DAPI$^-$TCRβ$^+$CD4$^+$) (**B**) and CD8 T cell (DAPI$^-$TCRβ$^+$CD8$^+$) (**C**) numbers in the spleen. (**D, E**) Representative flow cytometry plots showing expression of CD62L and CD44 within the splenic CD4 (DAPI$^-$TCRβ$^+$CD4$^+$ population) (**D**) and CD8 (DAPI$^-$TCRβ$^+$CD8$^+$ population) (**E**) T cells. (**F, H**) Total T cell (**F**), CD4 T cell (**G**) and CD8 T cell numbers in lymph nodes. (**I, J**) Representative flow cytometry plots showing expression of CD62L and CD44 within the CD4 (DAPI$^-$TCRβ$^+$CD4$^+$ population) (**I**) and CD8 (DAPI$^-$TCRβ$^+$CD8$^+$ population) in the lymph nodes (**J**).

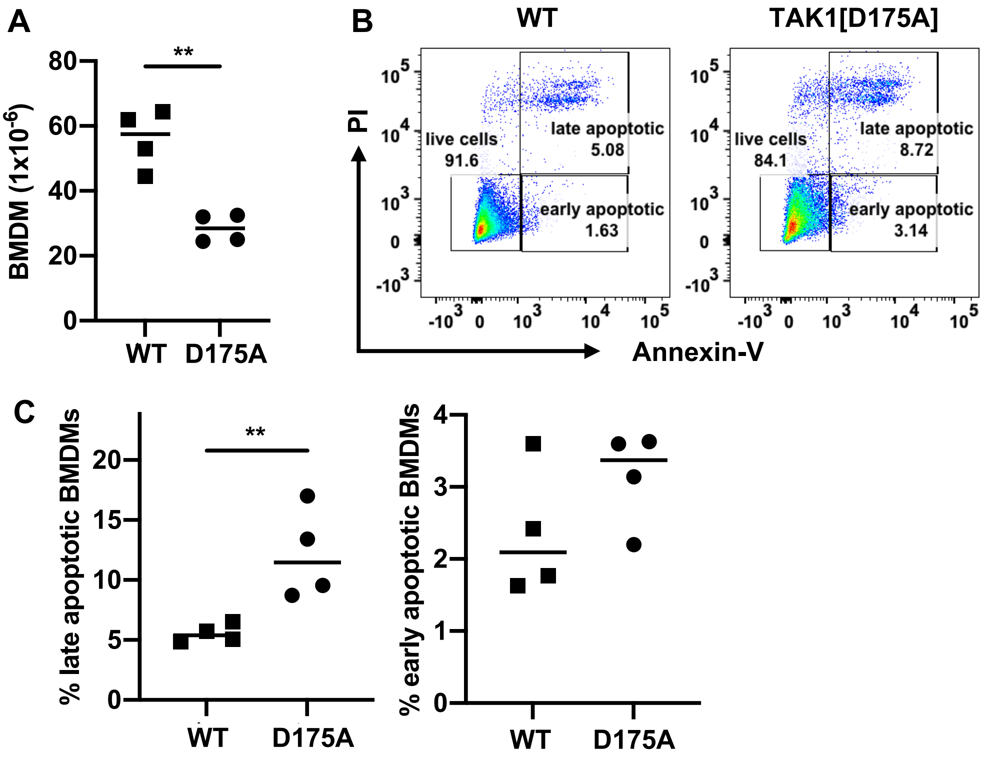

Supplementary Figure S3. Increased apoptosis and reduced production of BMDM from TAK1[D175A] x Vav-iCre mice.

(**A**) BMDM numbers obtained from the bone marrow of 4 WT and 4 TAK1[D175A] x Vav-iCre mice after 7 days culture in L929 medium. **(B**) Representative flow cytometry plots showing propidium iodide and Annexin-V staining in BMDM from from WT and TAK1[D175A] x Vav-iCre mice. (**C**) As in (**B**) except that the percentage of late (PI^+Annexin-V^+) and early (PI^- Annexin-V^+) apoptotic BMDM from 4 WT and 4 TAK1[D175A] x Vav-iCre mice are shown. The statistical analysis in A using the unpaired t-test with Welch's correction and in C was done using the Mann-Whitney test and; ** denotes p<0.01

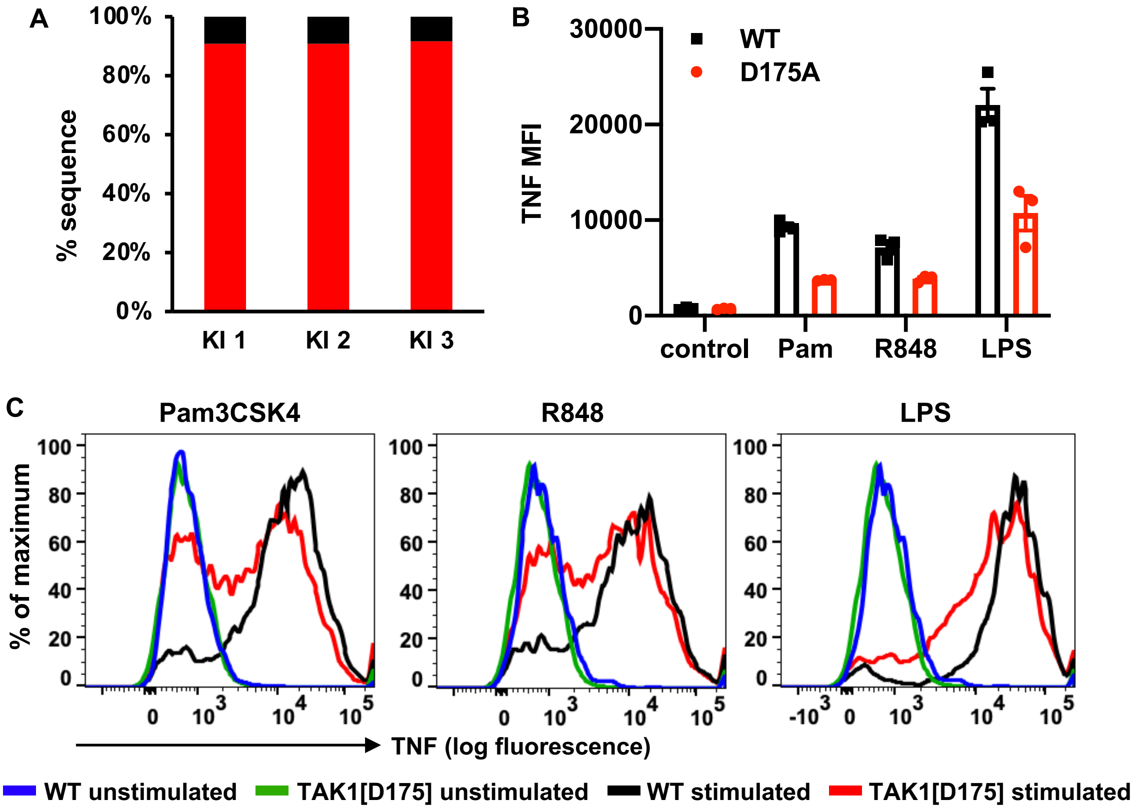

Supplementary Figure S4. Flow cytometry analysis of TNF production in BMDM from WT and TAK1[D175A] mice.

(**A**) RNA from the BMDM of three different conditional TAK1[D175A] knock-in mice (KI 1, KI 2 and KI 3) was analysed using TaqMan probes and the percentage of the D175A mutant (red bars) and WT TAK1 (black bars) was quantitated (see Methods). (**B)** To detect TNF production in individual BMDM from three WT (black symbols) and three TAK1[D175A] mice (red symbols), the cells were stimulated for 4 h with Pam3CSK4 (1μg/ml) (Pam), R848 (250 ng/ml) or LPS (100 ng/ml) in the presence of 5 mg/ml Brefeldin A (#420601, Biolegend) to prevent secretion or left unstimulated (control). BMDM were then stained with Fixable Viability Dye eFluor™ 450 (eBioscience™, ThermoFisher) according to the manufacturer's instructions and fixed for 20 min (IC fixation buffer #00-8222-49, ThermoFisher) prior to treatment with Permeabilization Buffer (#00-8333 ThermoFisher). The cells were blocked, stained with a TNF antibody (APC #506307, Biolegend diluted 1:200) and analysed by flow cytometry. Samples were processed using BD LSRFortessa or BD FACSCanto and the results analysed using FlowJo software (Tree star). The graph shows average values (± SEM) of median fluorescence intensity (MFI) of TNF staining. (**C**) As in B but showing representative flow cytometry plots of BMDM from one WT and one TAK1[D175A] mouse.

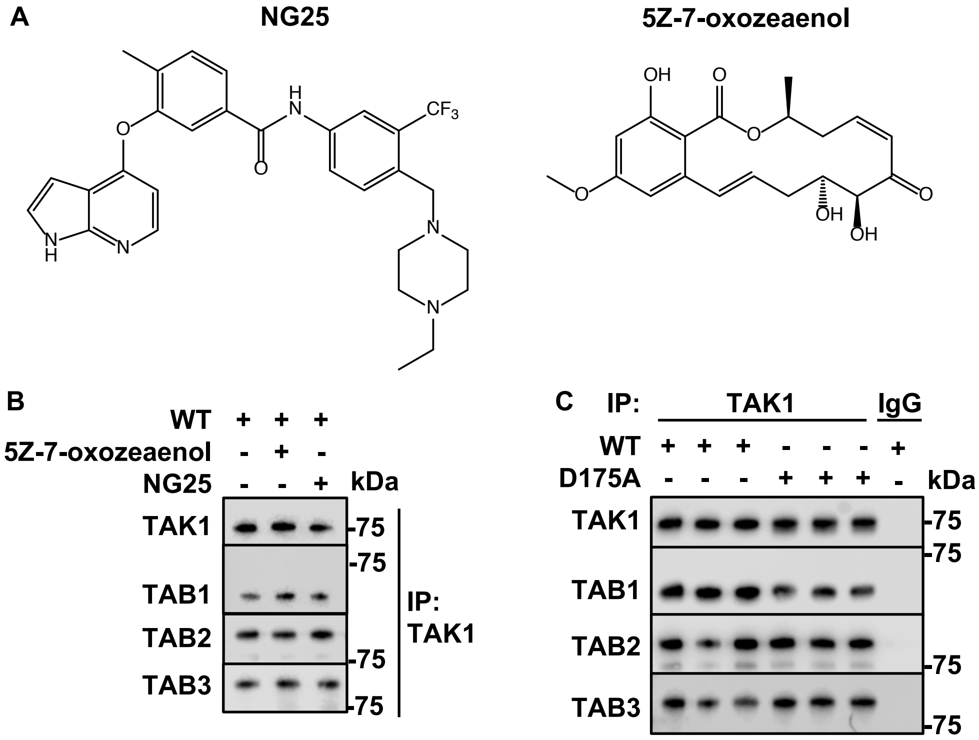

Supplementary Figure S5. The inhibition of TAK1 or its mutation does not cause dissociation of the regulatory TAB subunits from the catalytic subunit.

(A) Chemical Structures of the TAK1 inhibitors NG25 and 5Z-oxozeaenol (**B, C**) WT BMDM extracts (0.5 mg protein, 0.25 ml) were incubated for 16 h at 4°C with TAK1 antibody (2 μg) or control IgG (2 mg) coupled to Protein G-Dynabeads™ (ThermoFisher) (containing 1.2 mg protein G), with (+) or without (-) 5Z-7-oxozeanol (3 μM) or NG25 (2 μM). The samples were placed on a magnetic stand and the magnetic beads washed twice with 50 mM Tris/HCl pH 7.5, 1% (v/v) Triton containing 0.5 M NaCl, and twice with 50 mM Tris/HCl, pH 7.5, 1% (v/v) Triton without NaCl, The immunoprecipitated proteins were then eluted from the magnetic beads with SDS and immunoblotted for the proteins indicated. (**C**) As in (B), except that BMDM extracts from WT or TAK1[D175A] mice were used in the absence of TAK1 inhibitors and preimmune IgG (2μg) was included as a control**.** Each lane shows the results from a separate mouse.

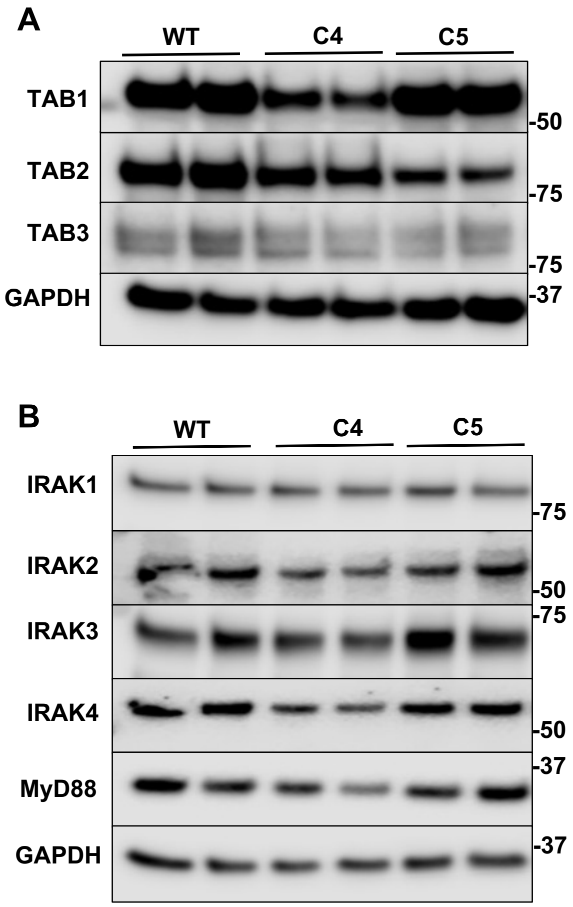

Supplementary Figure S6. Expression of the TAK1 regulatory subunits and components of the myddosome is similar in TAK1 KO and WT THP1 cell lines.
Cell extracts (10 mg) from WT and TAK1 KO THP1 cells (clones C4 and C5) were denatured in SDS, subjected to SDS/PAGE, followed by immunoblotting with antibodies recognizing TAB1, TAB2, TAB3 (**A**) and the myddosome components IRAK1, IRAK2, IRAK3, IRAK4 and MyD88 (**B**). Immunoblotting for GAPDH was carried out as a loading control.
